# Interactive effects of nitrogen and light on growth rates and RUBISCO content of small and large centric diatoms

**DOI:** 10.1007/s11120-016-0301-7

**Published:** 2016-08-26

**Authors:** Gang Li, Douglas A. Campbell

**Affiliations:** 1 Biology Department, Mount Allison University, Sackville, NB E4L 1G7 Canada; 2 Key Laboratory of Tropical Marine Bio-Resources and Ecology, South China Sea Institute of Oceanology, CAS, Guangzhou, 510301 China

**Keywords:** Cell size, Chlorophyll, Phytoplankton, Nitrogen metabolism, RUBISCO, *Thalassiosira pseudonana*, *Thalassiosira punctigera*

## Abstract

Among marine phytoplankton groups, diatoms span the widest range of cell size, with resulting effects upon their nitrogen uptake, photosynthesis and growth responses to light. We grew two strains of marine centric diatoms differing by ~4 orders of magnitude in cell biovolume in high (enriched artificial seawater with ~500 µmol L^−1^ µmol L^−1^ NO_3_^−^) and lower-nitrogen (enriched artificial seawater with <10 µmol L^−1^ NO_3_^−^) media, across a range of growth light levels. Nitrogen and total protein per cell decreased with increasing growth light in both species when grown under the lower-nitrogen media. Cells growing under lower-nitrogen media increased their cellular allocation to RUBISCO and their rate of electron transport away from PSII, for the smaller diatom under low growth light and for the larger diatom across the range of growth lights. The smaller coastal diatom *Thalassiosira pseudonana* is able to exploit high nitrogen in growth media by up-regulating growth rate, but the same high-nitrogen growth media inhibits growth of the larger diatom species.

**Electronic supplementary material:**

The online version of this article (doi:10.1007/s11120-016-0301-7) contains supplementary material, which is available to authorized users.

## Introduction

Nitrogen is critical for marine phytoplankton to build abundant cellular materials including proteins and nucleic acids to support their growth and cell division (Finkel et al. 2010; Wu et al. 2014b; Li et al. 2015). One of these nitrogen-rich materials the ribulose-1,5-bisphosphate carboxylase oxygenase (RUBISCO) protein is particularly important, because this bifunctional enzyme catalyzes the initial step of photosynthetic carbon reduction by combining CO_2_ with ribulose-1,5-bisphosphate (RuBP) (Mizohata et al. 2002), fixing inorganic carbon to organic matter (Kroth 2015). Nitrogen availability can affect the cellular content of RUBISCO with consequent effects upon phytoplankton carbon assimilation (Wilhelm et al. 2006), particularly under N-limited conditions where RUBISCO has been suggested to act as an N reservoir in some species (Falkowski et al. 1989). Available nitrogen is indeed considered as the proximal limiting factor for marine primary production (Gruber and Galloway 2008; Moore et al. 2013) in much of the modern ocean. At the other extreme, nitrogen compounds have diverse toxic effects, including inhibition of photosystem II (PSII) (Drath et al. 2008).

Marine diatoms are one of the dominant groups of phytoplankton (Wilhelm et al. 2006) in the modern ocean (Bowler et al. 2010) and account for approximately 20 % of global primary productivity (Field et al. 1998). Diatoms also span a wide size range across species from <2 µm to over 200 µm in equivalent spherical diameter, giving over eight orders of magnitude in cell volume (Beardall et al. 2009) Finkel et al., 2010). Diatom cell size affects many physiological processes, including light energy absorption (Finkel 2001; Key et al. 2010), photosynthesis and respiration (Wu et al. 2014a; López-Sandoval et al. 2014), nutrient diffusion and uptake (Raven 1998; Raven and Kübler 2002; Marañón et al. 2013), and ultimately affects their growth (Mei et al. 2011; Marañón et al. 2013; Wu et al. 2014a).

Small phytoplankton cells have a higher surface area-to-volume ratio and can have lower nutrient requirements for growth (Raven 1998; Finkel et al. 2010); thus, they often dominate in nitrogen-limited and clear oceanic waters (Clark et al. 2013). Furthermore, pigment packaging in large phytoplankton can lead to a decline in light absorption per unit of pigment–protein complex (Finkel 2001; Key et al. 2010; Clark et al. 2013), and thus, a decrease in captured photons per unit of metabolic nitrogen invested into pigment–protein complexes (Raven 1984; Wu et al. 2014b) and carbon assimilation per RUBSICO molecule (Wu et al. 2014b), again conferring advantages to smaller phytoplankton. The prevalence of larger phytoplankton cells in eutrophic and brackish coastal waters can be explained by enhanced resistance to predation (Ward et al. 2012), greater nutrient storage capacity (Grover 2011) and potentially lower metabolic costs to withstand and exploit fluctuating light because of lower susceptibility to photoinactivation of PSII (Key et al. 2010). We found that under near-saturating growth light and media with high-nitrogen larger diatoms invested more cellular nitrogen to RUBISCO than did smaller ones, to counter a lower achieved RUBISCO turnover rate (Wu et al. 2014b), a finding that parallels increased allocations to RUBISCO in cold water diatoms where RUBISCO performance is kinetically limited (Losh et al. 2013; Young et al. 2015). To explain our finding of slower achieved RUBISCO turnover in larger diatoms (Wu et al. 2014b), we hypothesized that in higher-nitrogen (HN) media large cells have luxury accumulation of RUBISCO protein that in turn lowers their achieved performance per unit RUBISCO. To test this hypothesis, we grew two representative diatom strains with a ~4 orders of magnitude difference in cell biovolume, small *Thalassiosira pseudonana* (~40 µm^3^) and large *T. punctigera* (~300,000 µm^3^) in turbidostats across a range of growth light, in lower-nitrogen (LN) media to attempt to limit cellular luxury accumulation of RUBISCO protein, compared with cells grown in a typical laboratory HN media. We determined the cellular nitrogen content and nitrogen allocations to chlorophyll *a* (Chl *a*), PSII and RUBISCO, as well as light capture and electron transport parameters of the small and large diatoms across the nitrogen levels and growth light ranges, and analyzed in parallel how changing RUBISCO contents and turnover rate interact with changes in growth rates.

## Materials and methods

### Culture protocol and growth rate

Two marine centric diatom strains *T. pseudonana* (CCMP 1335) and *Thalassiosira punctigera* (CCAP 1085/19) were obtained from the Provasoli–Guillard National Center of Marine Phytoplankton and cultured in rectangular cuvettes (450 mL volume) of FMT-150 photobioreactors with two-cm optical pathlength for illumination from a flat array of blue LED lights facing the rear face of the cuvette (Photon Systems Instruments, Drasov, Czech Republic) at 18 °C. We used a high-nitrogen media [HN, enriched artificial seawater (ESAW)] with ~550 µmol L^−1^ NO_3_^−^ (Berges et al. 2001), originally from Harrison et al. (1980), except with 54.5 µmol L^−1^ Si and 0.82 µmol L^−1^ Sr to limit precipitation during autoclaving. This HN media maintained ~500 µmol L^−1^ NO_3_^−^, in the face of cellular uptake in the turbidostat cultures. We also used a LN media (LN, ~55 µmol L^−1^ NO_3_^−^), the same ESAW except with one-tenth of the nitrogen level of HN media, corrected with equal sodium bicarbonate to equivalent total alkalinity. The LN media maintained ~5–10 µmol L^−1^ NO_3_^−^ in the turbidostat cultures. We gently mixed the cultures with a curtain of bubbles emitted from four apertures across the cuvette bottom with outdoor fresh air that was filtered through a 0.2-µm micro-filter and bubbled through sterile distilled water for humidification before bubbling through the culture cuvette. We provided continuous growth light measured with a microspherical quantum sensor (US-SQS, Walz, Germany). The light levels in the culture vessels filled with media were set to 30, 180 and 380 μmol photons m^−2^ s^−1^ for *T. pseudonana*, but to 30, 90 and 180 μmol photons m^−2^ s^−1^ for *T. punctigera*, because the *T. punctigera* growth was already inhibited by 180 μmol photons m^−2^ s^−1^, it would not grow under 380 μmol photons m^−2^ s^−1^ in our turbidostats with continuous light.

As described in detail for a previous, separate, project (Li and Campbell 2013), we grew each experimental replicate culture from initial inoculation for 5–6 generations without dilution in batch mode to reach a culture suspension density set point for subsequent turbidostat mode, which was monitored continuously using the onboard fluorescence sensor to track pigment content in the bioreactors. When the fluorescence value reached the set point, the bioreactors activated a peristaltic pump to dilute the 450-mL culture with a 10 % volumetric addition of media delivered from a reservoir, which was continuously pre-bubbled with a fresh air stream before adding to the cultures. We used cell counts (data not shown) to calibrate the fluorescence detector set point to control the culture cell suspension density to 3.6 ± 1.8 × 10^5^ cells mL^−1^ for *T. pseudonana* and 650 ± 110 cells mL^−1^ for *T. punctigera*. The variation in cell number across treatments (20–50 % SD) resulted mainly from changing pigment contents per cell across growth lights and thus differences in fluorescence signal per cell. We accepted a variation in cell number mL^−1^ across our treatments to keep the pigment-based bio-optical culture thickness and thus light attenuation, comparable across the treatments. Under these turbidostat growth conditions, the cells depleted the NO_3_^−^ concentration within the culture volume to ~500 µmol L^−1^ under HN and to <10 µmol L^−1^ under LN media. 3.5 L of media in the reservoir supported ~9.5 cellular generations of turbidostat growth for each replicate culture under the set conditions of light and nitrogen, before we harvested for physiological and biochemical analyses. Depending upon the achieved growth rate, which varied with growth light, nitrogen and species, the temporal duration of each turbidostat run ranged from 160 to 200 h. We calculated the specific growth rate for a given culture replicate by using the onboard detector to monitor optical density at 680 nm and then fitting the increase with time with an exponential growth function for the interval between each turbidostat 10 % dilution cycle. We then averaged the exponential rates from the final ten cycles of turbidostat dilution to estimate the growth rate for each turbidostat replicate for each growth condition (Li and Campbell 2013). We grew three or four separate turbidostat replicate cultures of each species under each combination of light and media, for a total of 39 separate turbidostat runs.

### Chlorophyll fluorescence measurements

At the end of each growth period we took out 2-mL culture from each turbidostat cuvette, incubated in darkness for 5 min within a cuvette with temperature control (18 °C) and measured the chlorophyll fluorescence with a fluorometer (PSI FL 3500, Photon Systems Instruments, Czech Republic) using the fast repetition rate fluorescence technique (Kolber et al. 1998). A series of 40 × 1.2 µs flashlets of blue light were applied over 128 μs (455 nm; ~100,000 µmol photons m^−2^ s^−1^) and were provided to progressively close PSII reaction centers. The resulting FRR induction curve was analyzed with the PSIWORX script for MATLAB (Audrey Barnett, www.sourceforge.net), to define *F*_0_, the base line fluorescence cells after 5 min darkness, F_M_, the maximum fluorescence with all PSII closed and *σ*_PSII_ (10^−20^ m^2^ quanta^−1^), the effective absorbance cross section serving PSII photochemistry in the dark-regulated cells. After a 2-s dark period, we applied a second FRR induction to test whether the initial FRR flashlet train perturbed the cells. We then activated an actinic blue light to the growth light level for 2 min; again, we captured the FRR induction curves to extract parameters in illuminated cells, to define *F*_S_, the fluorescence in light acclimated state, $F_{\text{M}}^{{\prime }} ,$ the maximum fluorescence with all PSII closed and $\sigma_{\text{PSII}}^{\prime }$ (10^−20^ m^2^ quanta^−1^), the effective absorbance cross section serving PSII photochemistry in the light acclimated state. We then applied an FRR induction after a 2-s dark period again, to allow reopening of closed PSII centers. There was a slight drop in the level of fluorescence from *F*_S_ to $F_{{ 0 2 {\text{s}}}}^{{\prime }} ,$ the baseline fluorescence with all PSII open but still in light acclimated state.

We calculated the maximum photochemical yield in dark-adapted cells as:

$$F_{\text{V}} /F_{\text{M}} = (F_{\text{M}} - F_{ 0} )/F_{\text{M}} ;$$

We also used the magnitude of any increase from $F_{\text{M}}^{{\prime }}$ to $F_{\text{M2s}}^{{\prime }}$ to apply a proportional correction to $F_{{ 0 2 {\text{s}}}}^{{\prime }}$ to estimate the actual level of $F_{ 0}^{{\prime }}$ under illumination:

$$F_{0}^{\prime } = F_{{02{\text{s}}}}^{\prime } \times \left\{ {1{-}\left[ {\left( {F_{{{\text{M}}2{\text{s}}}}^{\prime } - F_{\text{M}}^{\prime } } \right)/F_{{{\text{M}}2{\text{s}}}}^{\prime } } \right]} \right\}$$

We estimated the electron transport rate away from PSII following (Huot and Babin 2010) as:

$${\text{PSII}}\,\,{\text{electron}}\,\,{\text{transport}} = \sigma_{\text{PSII}}^{\prime } \times I \times q_{\text{P}} ,$$

where $\sigma_{\text{PSII}}^{\prime }$ (m^2^ quanta^−1^) is the effective absorbance cross section serving PSII photochemistry at growth light I (photons m^−2^ s^−1^) and *q*_P_(*F*′_M_ − *F*_S_)/(*F*′_M_ − *F*′_0_) is the proportion of PSII instantaneously open and ready to perform photochemistry under light I. We also used the FRR data and PSIWORX MATLAB to extract the life times for fluorescence relaxation after the saturating flashlet train, *τ*_1_ and *τ*_2_, to measure the downstream capacity to carry electrons away from PSII (Kolber et al. 1998).

### Cell density, protein and chlorophyll *a* measurements

At the end of each turbidostat growth period, we took duplicate 2-mL culture samples, fixed in Lugol’s acid solution and measured the cell suspension density with a Coulter Z2 counter (Beckman Instruments, FL, USA) for *T. pseudonana* and with a Sedgwick Rafter chamber under a microscope for *T. punctigera*. Simultaneously, we vacuum-filtered 50 mL of culture sample onto a binder-free Whatman GF/F glass fiber filter (25 mm in diameter), which was immediately flash frozen in liquid nitrogen and stored at −80 °C until later analyses of protein and chlorophyll.

Immunoquantitations of specific protein subunits from total protein extracts followed (Brown et al. 2008). Briefly, total protein was extracted from the frozen filters and 450 μL of 1× denaturing extraction buffer (0.1375 mol L^−1^ TRIS buffer, 0.075 mol L^−1^ LDS, 1.075 mol L^−1^ glycerol, 0.5 mmol L^−1^ EDTA, 0.1 mg mL^−1^ Pefabloc) (Brown et al. 2008) for three cycles of 60 s at 6.5 m s^−1^ using a MPBio FastPrep^®^-24 with bead lysing matrix D (SKU 116913050). Total protein in the extracts was determined using Bio-Rad DC protein assay kit (500-0116) with known bovine gamma globulin standards. Samples containing 0.5 µg of total protein were loaded on 4–12 % acrylamideprecast NuPAGE gels (Invitrogen) to determine RUBISCO large subunit (RbcL), run in parallel with a range of 1500, 750, 375 and 187.5 fmol RbcL standard per standard lane (Agrisera, www.agrisera.se, AS01 017S) to establish a standard curve. To determine PSII PsbA subunit, we loaded 1 µg of total protein per lane, run in parallel with a range of 125, 62.5, 31.25 and 15.625 fmol PsbA standard per standard lane (Agrisera, www.agrisera.se, AS01 016S). Electrophoresis was run for 25 min at 200 V and the proteins were transferred to a polyvinylidene fluoride (PVDF) membrane for 60 min at 30 V for RbcL and 20 V for PsbA. After the membrane blocking in 2 % ECL Advance blocking reagent (GE Healthcare) in 20 mmol L^−1^ Tris, 137 mmol L^−1^ sodium chloride (pH 7.6) with 0.1 % (v/v) Tween-20 (TBS-T) overnight at 4 °C, a primary antibody (Agrisera, AS03 037 and AS05 084, 1:20,000 in 2 % ECL Advance blocking dilution) was applied, followed by an anti-rabbit secondary antibody coupled with horseradish peroxidase (Agrisera, AS09 602, 1:20,000). The membranes were then developed by chemoluminescence using ECL Advance (GE Biosciences) and imaged under a CCD imager (BioRadVersaDoc 4000MP). Finally, the RbcL and PsbA protein quantitation was determined by fitting the sample signal values to the protein standard curves, taking care that all sample signals fell within the range of protein standard curve and that no band signals were saturated (Brown et al. 2008).

For Chl *a* measurement, 50 µL of protein extract was added to 450 µL 90 % acetone (v/v) saturated with magnesium carbonate; after 15 min extraction in the dark at 4 °C and 2 min centrifugation (13,000*g*), we measured the absorbance of the supernatant at 664, 630 and 750 nm using a UV/VIS photospectrometer (UV-1800, Shimadzu, Japan). The concentration of the Chl *a* in the extract (µg Chl *a* mL^−1^) was estimated following (Jeffrey and Humphrey 1975):

$$[{\text{Chl}}a] = 11.47 \times (A_{664} - A_{750} ) - 0.4 \times (A_{630} - A_{750} )$$

### Carbon and nitrogen analyses

At the end of each turbidostat growth period, we filtered 50 mL of culture onto a pre-combusted (5 h, 450 °C) Whatman GF/F glass fiber filter (13 mm in diameter), rinsed with 10 mL of 50 mmol HCl L^−1^ to remove inorganic carbon, dried at 55 °C for 12 h and stored in a desiccator for later analyses. Contents of carbon and nitrogen were measured with a Vario EL III Elemental Analyzer (Elementar, Hanau, Germany).

### Data analysis

The responses of growth rate to culture growth light were fitted with either linear regressions or the equation of Eilers and Peeters (1988):

$$\mu = I/\left( {a \times I^{2} + b \times I + c} \right)$$

where *µ* is growth rate (day^−1^) at the particular light level; I is the growth light (µmol photons m^−2^ s^−1^) and *a*, *b* and *c* are fitted parameters and

$$\mu_{\hbox{max} } = 1/\left( {b + 2\sqrt {a \times c} } \right).$$

We estimated apparent carbon assimilation per RUBISCO active site (C RbcL^−1^ s^−1^) as:

$$\begin{aligned} & {\text{RUBISCO}}\,{\text{turnover}}\,{\text{rate}}\, ( {\text{C}}\,{\text{RbcL}}^{ - 1} \,{\text{s}}^{ - 1} )\\ & \quad = ({\text{C}}\,{\text{cell}}^{ - 1} ) \times ({\text{e}}^{\mu } - \, 1) \times ({\text{cell}}\,{\text{RbcL}}^{ - 1} ) \\ \end{aligned}$$

We used ANOVA with Bonferroni posttests (Prism 5, Graphpad Software) and comparisons of linear and nonlinear curve fits to detect significant differences (*p* < 0.05) in parameters between cultures from LN and HN media of each species. In figures, we include the 95 % confidence intervals on regression fits. We only plot and report linear regressions with slopes significantly different from 0. We used *t* tests to detect significant differences (*p* < 0.05) between variables measured under LN and HN under each growth light.

## Results

**Fig. 1:**
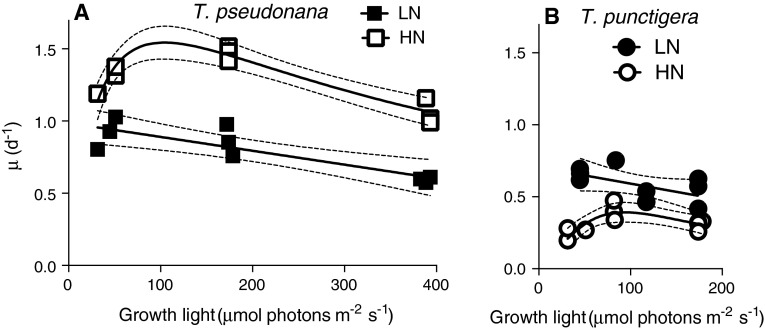
Growth rate (*µ*, day^−1^) versus culture growth light (µmol photons m^−2^ s^−1^) of the small diatom **a** *T. pseudonana* and **b** large diatom *T. punctigera* under low- (LN, *filled symbols*) and high-nitrogen media (HN, *open symbols*). *Solid lines* light response curves (Peeters and Eilers 1978; Eilers and Peeters 1988) or linear regressions; *thin dotted lines* show 95 % confidence intervals on the fitted curves. Note that there were small variations in the light sources among our three bioreactor units, so we plotted the specific growth rates and other parameters versus the actual light level applied to a given culture condition replicate, resulting in small offsets in data points along the *X* axes of plots, which slightly improved the statistical power of our curve fitting

The cell growth rate of *T. pseudonana* growing in HN media increased with growth light from low (~30 µmol photons m^−2^ s^−1^) to moderate levels (~180 µmol photons m^−2^ s^−1^) reaching a fitted *µ*_max_ of 1.5 day^−1^ but then decreased under higher light (Fig. 1a). Under LN 30 µmol photons m^−2^ s^−1^ was already growth-saturating for *T. pseudonana* (Fig. 1a), albeit at a lower *µ*_max_ than under HN (*p* < 0.05). The growth rate of *T. punctigera* in HN media was much slower than *T. pseudonana* (*p* < 0.05) but showed a qualitatively similar pattern of response to light, increasing with growth light from ~30 to ~90 µmol photons m^−2^ s^−1^ with a fitted *µ*_max_ of 0.39 day^−1^ but then decreasing under higher light (Fig. 1b). The growth rate of *T. punctigera* in LN media was significantly (*p* < 0.05) higher than in HN media, particularly under the lower growth light (Fig. 1b). For *T. punctigera* under LN 30 µmol photons m^−2^ s^−1^ was already growth-saturating, with a *µ*_max_ higher (*p* < 0.05) under LN than under HN.

The mass C:N ratio increased for both species with increasing growth light in LN media (*p* < 0.05), but not in HN media (Table 1). The only significant effect of media N upon C:N ratio was for *T. punctigera* growing at the highest growth light (*p* < 0.05). Chl *a* per cell decreased significantly (*p* < 0.05) with increasing growth light for both species in LN and in HN media, with no significant effects of media nitrogen (Table 1).

**Table 1:** Carbon per cell (pg cell^−1^), mass C:N ratio, and cellular contents of chlorophyll *a* (pg cell^−1^), total protein (pg cell^−1^), photosystem II PsbA subunit (amol cell^−1^) and RUBISCO large subunit (RbcL, amol cell^−1^) of *T. pseudonana* and *T. punctigera* under low- (LN) and high-nitrogen media (HN) across three levels of growth light

Strains	Growth light (µmol photons m^−2^ s^−1^)	Media N (µmol L^−1^)	Carbon (pg cell^−1^)	C:N	Chl*a* (pg cell^−1^)	Total protein (pg cell^−1^)	PsbA (amol cell^−1^)	RbcL (amol cell^−1^)
*T. pseudonana*	30	55	**10**.**5** **±** **0**.**52**	6.25 ± 0.20	0.25 ± 0.03	**7**.**12** **±** **0**.**65**	**0**.**73** **±** **0**.**08**	**21**.**2** **±** **0**.**91**
		550	**8**.**29** **±** **0**.**84**	5.89 ± 0.50	0.27 ± 0.07	**4**.**88** ± 0.73	0.30 ± 0.04	7.23 ± 0.92
	180	55	9.25 ± 2.00	6.41 ± 0.12	0.20 ± 0.04	4.49 ± 0.41	0.43 ± 0.03	8.68 ± 1.44
		550	8.09 ± 0.48	6.42 ± 0.32	0.13 ± 0.03	4.61 ± 0.23	0.33 ± 0.07	8.30 ± 1.45
	380	55	8.91 ± 1.41	7.67 ± 0.52	0.08 ± 0.02	4.31 ± 1.28	0.40 ± 0.07	8.25 ± 1.52
		550	8.71 ± 0.87	6.34 ± 0.87	0.09 ± 0.01	4.64 ± 0.37	0.34 ± 0.05	10.0 ± 2.55
*T. punctigera*	30	55	5669 ± 560	5.30 ± 0.64	142 ± 37.6	4424 ± 1221	246 ± 50.7	9578 ± 5547
		550	7675 ± 430	4.82 ± 0.32	115 ± 5.31	5703 ± 893	230 ± 64.2	3524 ± 777
	90	55	6752 ± 434	7.61 ± 2.10	83.5 ± 21.3	3992 ± 1192	215 ± 103	5178 ± 2128
		550	6921 ± 186	5.24 ± 0.50	79.1 ± 24.1	4475 ± 778	208 ± 44.6	3138 ± 530
	180	55	8281 ± 1421	9.28 ± 1.88	59.4 ± 28.5	4225 ± 54.3	206 ± 82.3	6977 ± 3219
		550	7160 ± 1565	5.20 ± 0.59	58.3 ± 14.8	3503 ± 430	168 ± 60.8	2657 ± 420

The values are presented as mean ± SD (*n* = 3 or 4); bold numbers show significant differences in cultures from LN and HN conditions (*p* < 0.05)

In *T. pseudonana*, total cellular protein content increased significantly (*p* < 0.05) under low media nitrogen and the lowest growth light. In *T. punctigera*, the protein content showed scatter among replicates, but showed a significant decrease under HN at the highest growth light (*p* < 0.05) (Table 1).

In *T. pseudonana*, the molar contents of PsbA subunit decreased in LN media with increasing growth light (*p* < 0.05). In the larger *T. punctigera*, PsbA contents decreased with increasing growth light in HN media (*p* < 0.05) (Table 1). The molar contents of RbcL subunit in HN media increased with increasing growth light for *T. pseudonana* but decreased for *T. punctigera* as growth light increased (Table 1). High-N media significantly decreased the RbcL content of *T. pseudonana* at the lowest growth light and that of *T. punctigera* at all growth lights when compared to LN media (*p* < 0.05).

**Fig. 2:**
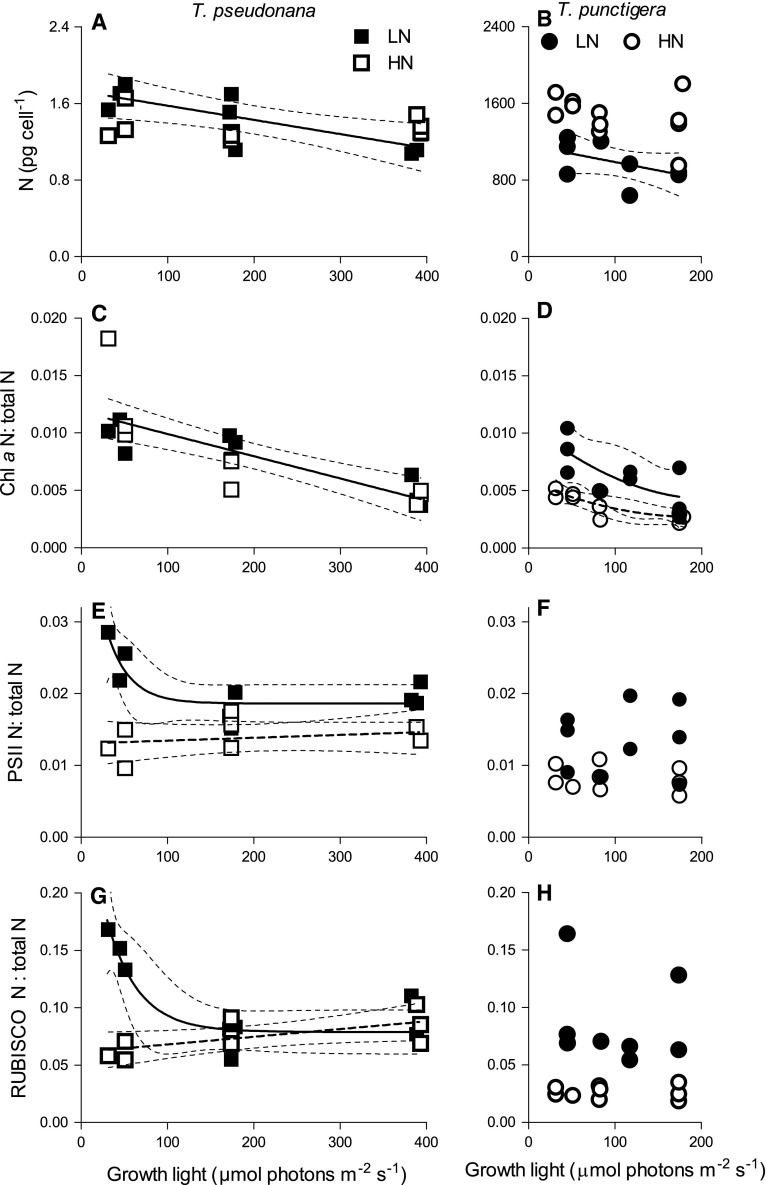
Nitrogen per cell (pg cell^−1^) versus culture growth light (µmol photons m^−2^ s^−1^) of **a** *T. pseudonana* and **b** *T. punctigera* under low- (LN, *filled symbols*) and high-nitrogen media (HN, *open symbols*). *Solid line* in **a**: linear regression for growth light response for *T. pseudonana* from LN condition only. *Solid line* in **b**: linear regression for growth light response for *T. punctigera* from LN condition only; *thin dotted lines* show 95 % confidence intervals on the fitted curves. Molar fraction of nitrogen allocated to chlorophyll *a* relative to total cellular nitrogen (Chl *a* N:total N) versus culture growth light (µmol photons m^−2^ s^−1^) of **c** *T. pseudonana* and **d** *T. punctigera*. *Solid line* in **c**: linear regression for pooled growth light response for *T. pseudonana* from LN and HN. *Lines* in **d**: polynomial curves for *T. punctigera* from LN (*solid*) and HN (*dashed*). Molar PSII nitrogen-to-total nitrogen ratio (PSII N:total N) versus culture growth light (µmol photons m^−2^ s^−1^) of **e** *T. pseudonana* and **f** *T. punctigera*. *Lines* in **e**: exponential decay for *T. pseudonana* from LN (*solid*) or linear regression from HN (*dashed*). Molar RUBISCO N:total N ratio versus culture growth light (µmol photons m^−2^ s^−1^) of **g** *T. pseudonana* and **h** *T. punctigera*. *Lines* in **g**: exponential decay for *T. pseudonana* from LN (*solid*) and linear regression from HN (*dashed*)

Cellular nitrogen content in *T. pseudonana* and in *T. punctigera* decreased significantly (*p* < 0.05) as growth light increased in LN media, but not under HN media (Fig. 2a, b). The low-N media also lowered *T. punctigera* cellular N content across all growth light conditions compared to HN (*p* < 0.05) (Fig. 2b). We estimated the fraction of cellular nitrogen allocated to Chl *a* (Table 1), to PSII (extrapolated from molar contents of the PsbA subunit, Table 1) and to RUBISCO protein (extrapolated from molar contents of the RbcL subunit, Table 1) as in Li et al. (2015). The molar fraction of Chl *a* N to total cellular N (Chl *a* N:total N) in *T. pseudonana* decreased with increasing growth light (*p* < 0.05), with no significant effect of media N (Fig. 2c). Under high nitrogen, the Chl *a* N:total N in *T. punctigera* decreased with increasing growth light (*p* < 0.05), and was also significantly increased by lower media N across the growth lights (*p* < 0.05) (Fig. 2d). The PSII N:total N in *T. pseudonana* increased under low media N at lowest growth light (Fig. 2e). The PSII N:total N in *T. punctigera* varied among replicates but with no apparent trends with growth light nor with media nitrogen level (Fig. 2f). The molar fraction of N allocated to RUBISCO relative to total cellular N under high nitrogen increased slightly for *T. pseudonana* as growth light increased, but showed no clear trends for *T. punctigera* (Fig. 2g, h). For *T. pseudonana* under low growth light, and for *T. punctigera* across the growth lights low-nitrogen media resulted in increased cellular content of RUBISCO, with a parallel increase in cellular protein content under high light. Thus, our initial goal of using lower media N to restrict the cellular allocation of N to RUBISCO failed for both species. Our results do show that in *T. punctigera* the RUBISCO content under HN does not likely represent luxury accumulation, because the fractional allocation of N to RUBISCO increased further under LN media.

**Fig. 3:**
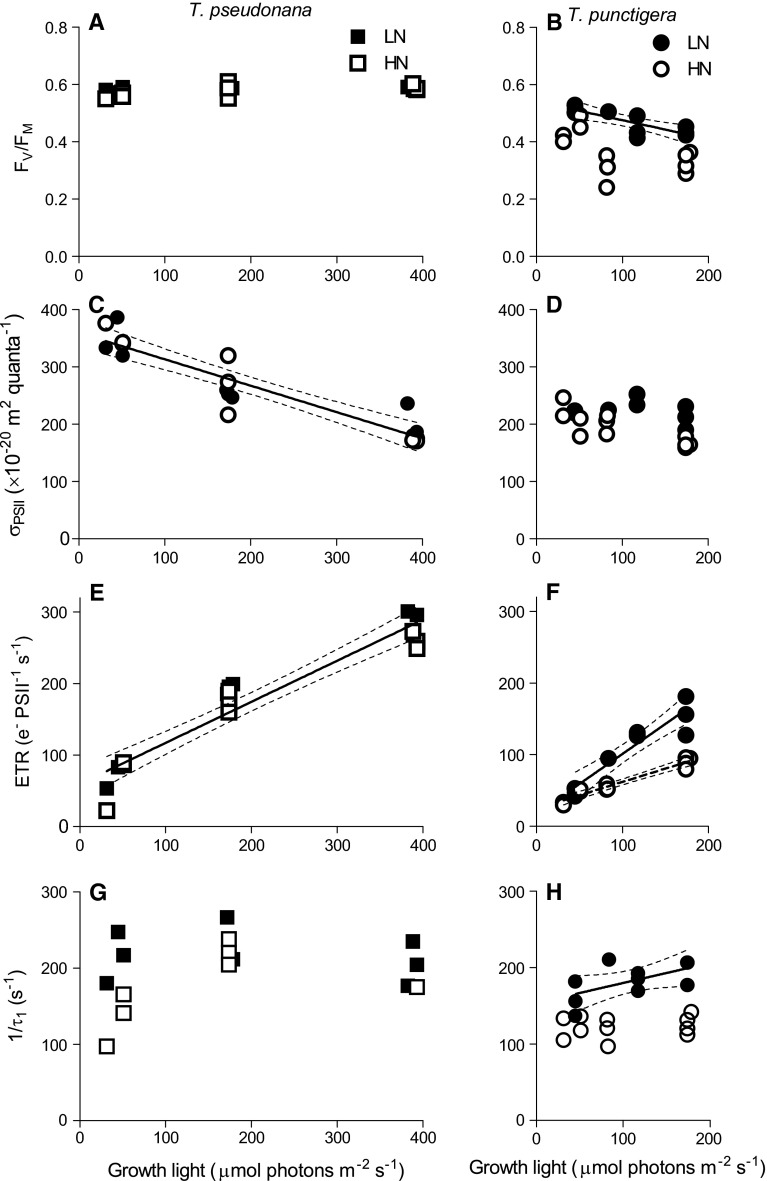
Maximum photosystem II photochemical quantum yield (*F*_V_/*F*_M_) versus culture growth light (µmol photons m^−2^ s^−1^) of **a** *T. pseudonana* and **b** *T. punctigera* under low- (LN, *filled symbols*) and high-nitrogen media (HN, *open symbols*). *Solid line* in **b**: linear regression for growth light response for *T. punctigera* from LN condition; thin *dotted lines* show 95 % confidence intervals on the fitted curves. Functional absorption cross section for PSII photochemistry (*σ*_PSII_, 10^−20^ m^2^ quanta^−1^) versus growth light (µmol photons m^−2^ s^−1^) of **c** *T. pseudonana* and **d** *T. punctigera*. *Solid line* in **c**: linear regression for pooled growth light response for *T. pseudonana* from LN and HN conditions. Electron transfer rate (e^−^ PSII^−1^ s^−1^) versus growth light (µmol photons m^−2^ s^−1^) of **e** *T. pseudonana* and **f** *T. punctigera*. *Solid line* in **e**: linear regression for pooled growth light response for *T. pseudonana* from LN and HN conditions. *Lines* in **f**: linear regressions for growth light response for *T. punctigera* from LN (*solid*) and HN (*dashed*) conditions. Slow decay lifetime (*τ*_1_, µs) of chlorophyll fluorescence versus culture growth light (µmol photons m^−2^ s^−1^) of **g** *T. pseudonana* and **h** *T. punctigera*. *Solid line* in **g**: light response curve (Eilers and Peeters 1988) for pooled *τ*_1_ from LN and HN conditions. *Solid line* in **h**: linear regression for growth light response for *T. punctigera* from LN condition

Maximum photochemical quantum yield of PSII (*F*_V_/*F*_M_) was ~0.58 in *T. pseudonana* with no significant effect of growth light, nor of media nitrogen level (Fig. 3a). The *F*_V_/*F*_M_ of *T. punctigera*, in contrast, decreased from 0.44 to 0.32 with increasing growth light in high-nitrogen media, but was somewhat higher in low-nitrogen media (Fig. 3b), showing that the high nitrogen resulted in a sustained down-regulation of PSII function, even under low growth light in *T. punctigera.* The functional absorption cross section for PSII photochemistry (*σ*_PSII_) in *T. pseudonana* decreased with increasing growth light, with no significant nitrogen effects (Fig. 3c), and showed a positive correlation with Chl *a* per cell (Table 1; Supplemental Figure 1, *R* ^2^ = 0.73). The *σ*_PSII_ for *T. punctigera* showed no significant effects of growth light nor of media nitrogen level (Fig. 3d). PSII electron transfer rate (ETR) for *T. pseudonana* increased with increasing growth light from 30 to 380 µmol photons m^−2^ s^−1^ (*R* ^2^ = 0.92), with no significant effect of media nitrogen (Fig. 3e). The PSII ETR for *T. punctigera* increased with growth light under low-nitrogen media, but showed a lower response to increasing light under HN media (Fig. 3f) because of lower PSII quantum yield and lower photochemical quenching (*q*_P_) under HN.

The rate constant for (1/*τ*_1_, s^−1^) for PSII reopening after a saturating flash reflects the kinetics of QA-re-oxidation (Kolber et al. 1998) and thus the capacity for downstream metabolism to balance PSII photochemistry. 1/*τ*_1_ in *T. pseudonana* showed no statistically significant effects of growth light, nor of media nitrogen level at our level of replication (Fig. 3g). 1/*τ*_1_ for *T. punctigera* was significantly (*p* < 0.05) higher in LN media, than in HN media (Fig. 3h), and accelerated with increasing growth light in LN media, but not in HN media. Thus, in *T. punctigera* the HN media decreased the capacity for electron transport away from PSII, thereby explaining the lower PSII ETR (Fig. 3b, h).

**Fig. 4:**
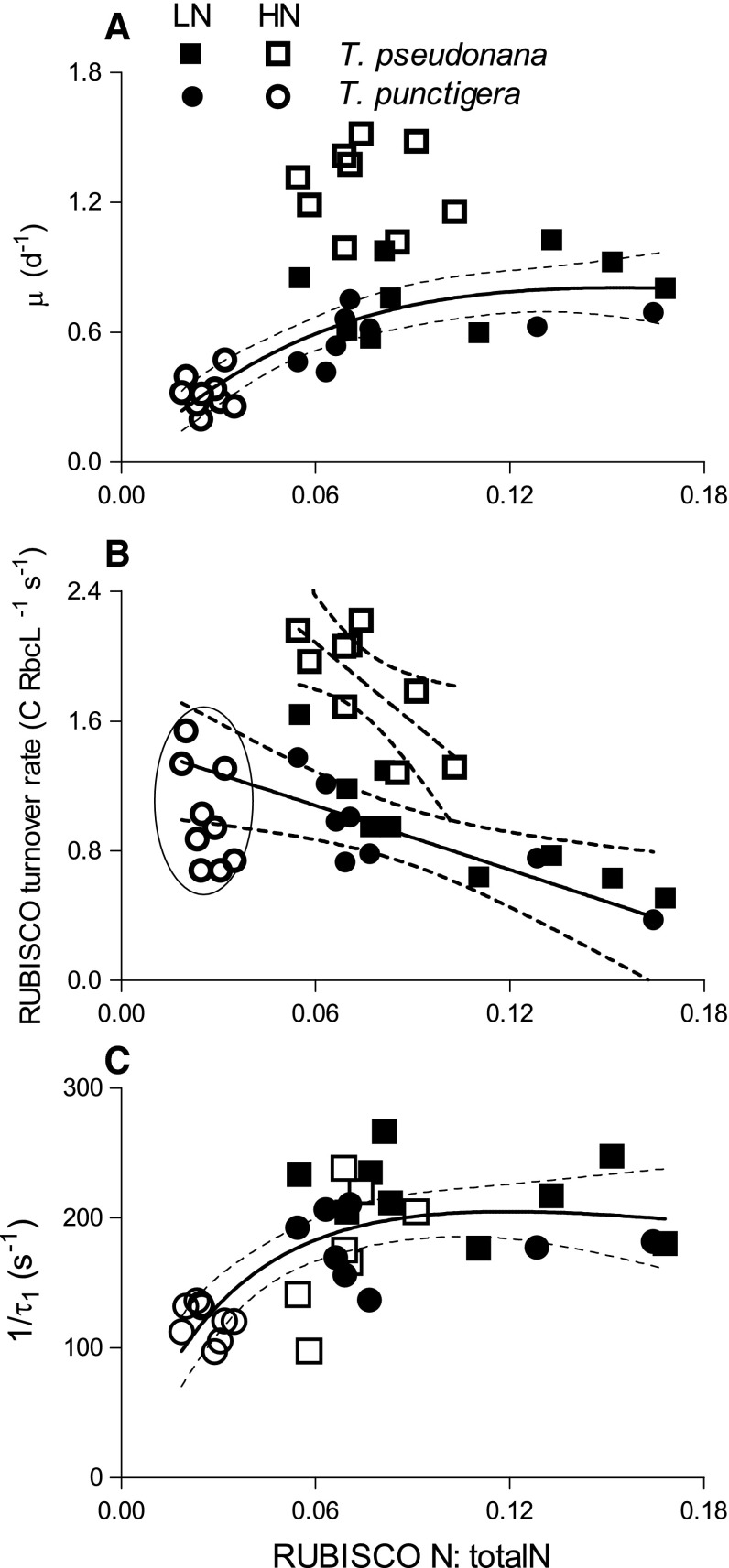
**a** Growth rate (*µ*, day^−1^) versus molar RUBISCO N-to-total cellular N ratio (RUBISCO N:total N) of *T. pseudonana* (*squares*) and *T. punctigera* (*circles*) under low- (LN, *filled symbols*) and high-nitrogen media (HN, *open symbols*). A common saturating function (Eilers and Peeters 1988) was fit to pooled data points, except *T. pseudonana* HN (*open squares*); *thin dotted lines* 95 % confidence intervals on the fitted curves. **b** Apparent RUBISCO turnover rate (C RbcL^−1^ s^−1^) versus RUBISCO N:total N of *T. pseudonana* and *T. punctigera*. *Solid line* linear regression of pooled C RbcL^−1^ s^−1^ versus RUBISCO N:total N for *T. pseudonana* from LN condition and *T. punctigera* from LN and HN conditions. *Oval* outlines *T. punctigera* HN measures, which were included in the pooled regression. *Dashed line* shows separate regression for *T. pseudonana* HN (*open squares*). **c** 1/*τ*_1_ (s^−1^), the rate of electron transport away from PSII, versus RUBISCO N:total N of *T. pseudonana* and *T. punctigera*. *Solid line* the best response curve fitted with (Peeters and Eilers 1978; Eilers and Peeters 1988)

**Fig. 5:**
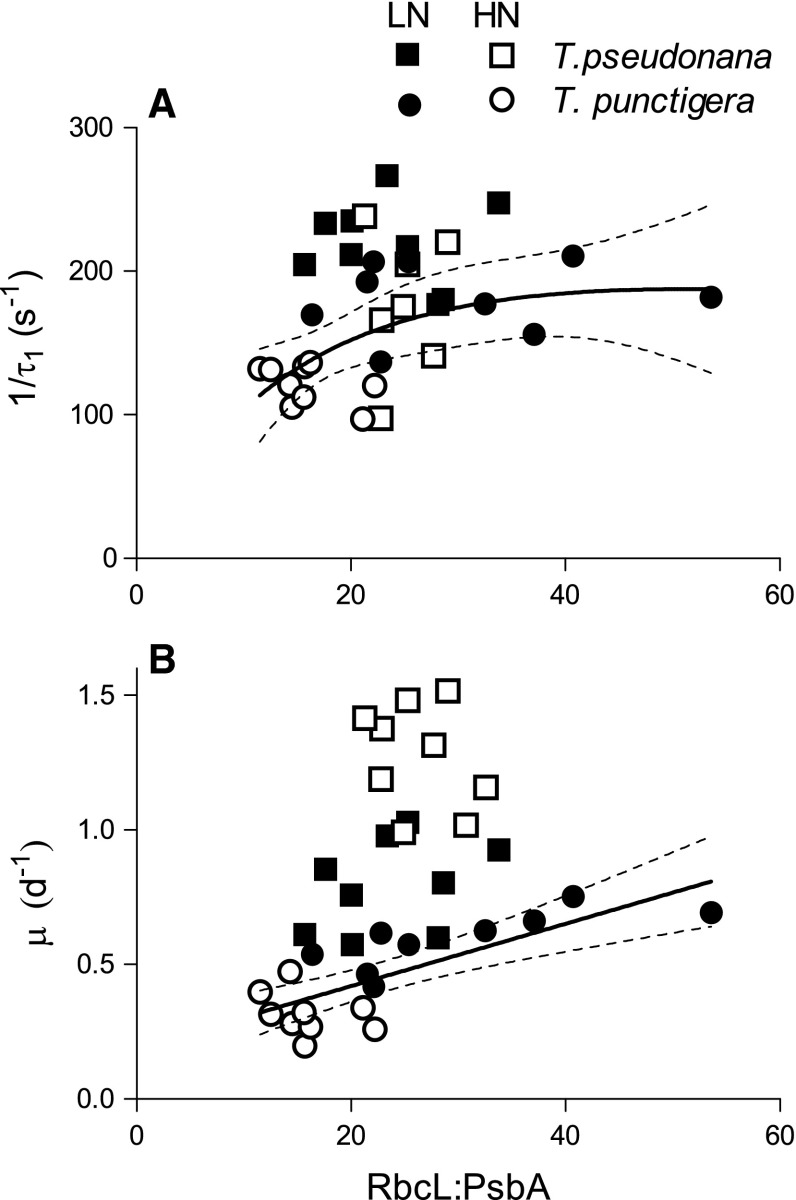
**a** 1/*τ*_1_ (s^−1^), the rate of electron transport away from PSII versus RbcL:PsbA ratio of *T. pseudonana* (*squares*) and *T. punctigera* (*circles*) under low- (LN, *filled symbols*) and high-nitrogen media (HN, *open symbols*). *Solid lines* the best response curve fit (Peeters and Eilers 1978; Eilers and Peeters 1988) for *T. punctigera*; *thin dotted lines* show 95 % confidence intervals on the fitted curves. **b** Growth rate (*µ*, day^−1^) versus RbcL:PsbA ratio of *T. pseudonana* and *T. punctigera* under LN and HN conditions. *Solid line* linear regression of pooled *µ* versus RbcL:PsbA for *T. punctigera* from LN and HN; *thin dotted lines* 95 % confidence intervals on the fitted curves

When we plotted growth rate (*µ*) against the nitrogen allocation to RUBISCO relative to total cellular nitrogen, a common, saturating, fit (Eilers and Peeters 1988) explained the variation in growth rates for both *T. pseudonana* (LN) and *T. punctigera* (LN, HN) (*R* ^2^ = 0.67), with growth rate reaching half saturation at a RUBISCO N:total N of 0.058. *T. pseudonana* grown in HN media, however, fell well above this pooled fitted curve (Fig. 4a), achieving higher growth rates at a given allocation RUBISCO N:total N. Similarly, the apparent RUBISCO turnover rate of *T. pseudonana* from LN media and *T. punctigera* from LN and HN media showed a negative linear correlation with RUBISCO N:total N (*R* ^2^ = 0.87); but again *T. pseudonana* achieved a higher apparent RUBISCO turnover rate in HN media (Fig. 4b). 1/*τ*_1_ shows a common saturating pattern when plotted versus RUBISCO N:total N (*R* ^2^ = 0.48), for both species across all treatments (Fig. 4c). On a plot of 1/*τ*_1_ versus RbcL:PsbA ratio, there was a saturating pattern for all treatments of *T. punctigera* (*R* ^2^ = 0.37), but not for *T. pseudonana* (Fig. 5a). *µ* versus RbcL:PsbA showed a linearly increasing trend for *T. punctigera* (*R* ^2^ = 0.59) (Fig. 5b).

## Discussion

Wu et al. (2014b) found that, compared to small diatoms, larger diatoms had a higher allocation of cellular nitrogen to RUBISCO, but lower achieved carbon assimilation per RUBISCO. Those findings were from diatoms growing semi-continuously in flasks under *f*/2 media (~850 µmol L^−1^ NO_3_^−^) and 350 µmol photons m^−2^ s^−1^ light with a 12:12 Light:Dark cycle. In this study we initially sought to test whether RUBISCO allocation in larger diatom cells represented a luxury accumulation of excess RUBISCO that could account for a low apparent RUBISCO turnover rate (Wu et al. 2014b). We therefore grew these same smaller and larger diatoms as continuous turbidostat cultures using photobioreactors to supply enriched artificial seawater at either HN, 550 µmol L^−1^ NO_3_^−^, or LN, 55 µmol L^−1^ NO_3_^−^, under a range of continuous light levels.

We achieved the expected growth limitation by lower culture nitrogen in the smaller *T. pseudonana*, which nevertheless maintained its RbcL content under low nitrogen (Table 1) and thus suffered a decrease in achieved RUBISCO turnover rate (Fig. 4b, closed vs. open squares) under low-nitrogen conditions. In contrast, at the lower culture nitrogen level (<10 µmol L^−1^ NO_3_^−^) the larger *T. punctigera* supported increased growth rates, particularly under lower light (Fig. 1b), although *T. punctigera* failed to grow under yet lower conditions of approximately <5 µmol L^−1^ NO_3_^−^ (data not presented). Therefore, we failed to achieve a reliably growth-limiting N concentration for *T. punctigera*.

Under LN, the larger diatom *T. punctigera* actually increased its cellular nitrogen allocation to RUBISCO (Fig. 2h; Table 1) across the range of growth lights, while in parallel achieving a RUBISCO turnover rate comparable to the rates achieved under HN (Fig. 4b). Thus, the low performance of RUBISCO turnover rate in our larger diatoms in (Wu et al. 2014b) was not attributable to luxury accumulation of excess RUBISCO, but rather to a limitation on their growth rates under high concentrations of NO_3_^−^ in typical laboratory media. These differences between studies illustrate the complexities of macromolecular allocations to cellular functions across strains and growth conditions (Young et al. 2016). The different L:D cycles between (Wu et al. 2014a) (12:12 L:D) and herein (24:0 L:D) could also contribute to the differences between the studies as shorter photoperiods increase the achieved RUBISCO turnover rate, particularly for larger diatoms (Li and Campbell, unpubl.).

Low N often limits phytoplankton growth (Mei et al. 2011; Moore et al. 2013; Li and Gao 2014) as indeed found here for *T. pseudonana* (Fig. 1a). There are two main strategies for phytoplankton to maintain growth rate when the growth environment is not favorable, to increase the abundance of rate-limiting enzymes or to increase the achieved enzymatic rates, as shown in particular by studies of the enzyme responsible for carbon fixation, RUBISCO (Feller and Gerday 2003; Young et al. 2015, 2016). Phytoplankton can thus allocate an increased fraction of their cellular N to growth rate-limiting enzymes, as for example RUBISCO, to maintain their growth when the media N source is low, consistent with our results for *T. pseudonana* grown at low growth light (Fig. 2g) and *T. punctigera* grown across three light levels (Fig. 2h). Meanwhile, the lower N source greatly decreased the achieved activity of RUBISCO in the small *T. pseudonana* but not in the larger *T. punctigera* (Fig. 4b), indicating differential effects of lower N source on both the amount and the achieved activity of RUBISCO in the smaller and larger diatom.

In analogy, Young et al. (2015) found that to compensate for slow enzymatic rates at low temperatures, Antarctic diatoms up-regulated both RUBISCO allocation and other cellular proteins needed for C fixation, to maintain productivity to support the intense spring/summer blooms in high-latitude waters, despite water temperatures close to freezing. Our results are consistent since higher RUBISCO abundance supported higher growth rates of both *T. pseudonana* and *T. punctigera*, but the relation shifted differentially for each species in high- or low-N media (Fig. 4a). *T. pseudonana*, but not *T. punctigera*, was able to up-regulate achieved RUBISCO performance to exploit higher levels of nitrogen in the media. Low-nitrogen media up-regulated the nitrogen allocation to RUBISCO rather than to PSII for *T. punctigera*, resulting in a higher RbcL:PsbA ratio (Fig. 5b). This in turn led to faster electron transport away from PSII (Fig. 5a) (Zorz et al. 2015), which might have ameliorated PSII photoinhibition and stimulated the cell growth (Fig. 5b) under LN.

In recent decades, coastal or estuarine nitrogen concentration has increased globally, as in Hong Kong waters where nitrate reaches 40 µmol L^−1^ in summer (Xu et al. 2012), in the Danube River estuary, northwestern Black Sea, where nitrate reached 50 µmol L^−1^ (Möbius and Dähnke 2015) and in the Jiulong River estuary, northern South China Sea, where nitrate reached over 80 µmol L^−1^ (Li et al. 2011). According to our findings (Fig. 1b), such nitrate levels are already well above the optimal growth nitrogen levels for large *T. punctigera*. In contrast, the smaller *T. pseudonana* can successfully exploit yet higher nitrate levels to achieve higher growth rates (Fig. 1a).

## Summary

We grew small and large marine centric diatoms, *T. pseudonana* and *T. punctigera*, in high- and low-nitrogen media, across a range of growth light. Growth-limiting LN media did not significantly lower cellular N content in the small *T. pseudonana*. In contrast, the same LN media stimulated faster growth in the larger *T. punctigera*, which actually increased cellular nitrogen content under low-nitrogen media compared to HN media. Furthermore, we found low-nitrogen media increased the cellular RUBISCO content of the smaller *T. pseudonana* at limiting light, but that *T. pseudonana* was able to up-regulate RUBISCO turnover under higher media N. The larger *T. punctigera* showed higher RUBISCO content under low-nitrogen media, in parallel with an increased capacity to carry electrons away from PSII. The larger *T. punctigera* showed sustained down-regulation of PSII function under high nitrogen (Drath et al. 2008), which was released in parallel with the increase in RUBISCO content under LN, providing a possible mechanism for the differential growth responses between the two diatoms.

### Data archiving

The data for this project are available through the database of Dr. Douglas A. Campbell Lab, phytoplankton.mta.ca.

## Electronic supplementary material

Below is the link to the electronic supplementary material.

Supplemental Figure: Functional absorption cross section for PSII photochemistry (σ_PSII_, 10^−20^ m^2^ quanta^−1^) versus Chl *a* content (pg cell^−1^) of A) *T. pseudonana* (squares) and B) *T. punctigera* (circles) under low- (LN, filled symbols) and high-nitrogen media (HN, open symbols). Solid line in panel A: Linear regression of pooled σ_PSII_ versus Chl *a* for *T. pseudonana* from LN and HN conditions; thin dotted lines: 95 % confidence intervals on the fitted curve. 1 (PDF 39 kb)
