# Supplementary figures and images for: Interactive effects of nitrogen and light on growth rates and RUBISCO content of small and large centric diatoms
Source: Photosynth Res. 2016 Aug 26;131(1):93–103. doi: 10.1007/s11120-016-0301-7 (PMC5167766; doi:10.1007/s11120-016-0301-7)

Figure Supplement

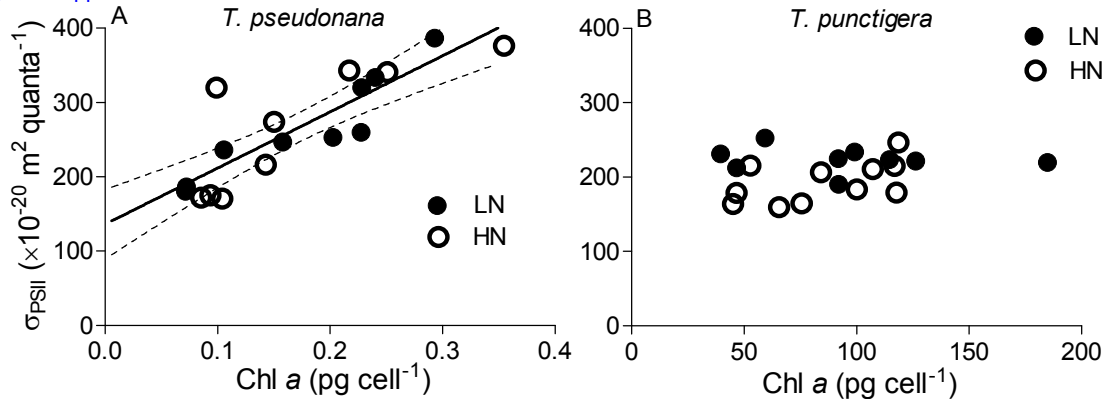

Supplement: Supplementary file 1 — Functional absorption cross section for PSII photochemistry (σPSII, 10−20 m2 quanta−1) versus Chl a content (pg cell−1) of A) T. pseudonana (squares) and B) T. punctigera (circles) under low- (LN, filled symbols) and high-nitrogen media (HN, open symbols). Solid line in panel A: Linear regression of pooled σPSII versus Chl a for T. pseudonana from LN and HN conditions; thin dotted lines: 95 % confidence intervals on the fitted curve. 1 (PDF 39 kb) [file 11120_2016_301_MOESM1_ESM.pdf]
